# Factors Associated with Satisfaction of Hospital Physicians: A Systematic Review on European Data

**DOI:** 10.3390/ijerph15112546

**Published:** 2018-11-13

**Authors:** Alicja Domagała, Małgorzata M. Bała, Dawid Storman, Juan Nicolás Peña-Sánchez, Mateusz J. Świerz, Mateusz Kaczmarczyk, Monika Storman

**Affiliations:** 1 Institute of Public Health, Faculty of Health Sciences, Jagiellonian University Medical College, 31-531 Krakow, Poland; 2 Chair of Epidemiology and Preventive Medicine, Department of Hygiene and Dietetics, Faculty of Medicine, Jagiellonian University Medical College, 31-034 Krakow, Poland; 3 Students’ Scientific Group of Systematic Reviews, Systematic Reviews Unit—Polish Cochrane Branch, Jagiellonian University Medical College, 31-034 Krakow, Poland; dawstor@wp.pl (D.S.); mefiu11@onet.eu (M.J.Ś.); mati@sudety.info.pl (M.K.); 4 Department of Community Health and Epidemiology, College of Medicine, University of Saskatchewan, Saskatoon, SK S7N5E5, Canada; juan.nicolas.ps@usask.ca; 5 Systematic Reviews—Polish Cochrane Branch, Jagiellonian University Medical College, 31-034 Krakow, Poland monika.storman@wp.pl; 6 Department of Diabetology and Internal Medicine, Medical University of Warsaw, 02-097 Warszawa, Poland

**Keywords:** physician satisfaction, job satisfaction, work environment, hospitals, factors associated

## Abstract

Background: Physician satisfaction is a multidimensional concept related to many factors. Despite the wide range of research regarding factors affecting physician satisfaction in different European countries, there is a lack of literature reviews analyzing and summarizing current evidence. The aim of the article is to synthetize the literature studying the factors associated with physician satisfaction. Methods: We searched: MEDLINE, Embase, PsycINFO, CINAHL and the Cochrane Library from January 2000 to January 2017. The eligibility criteria included: (1) target population: physicians working in European hospitals; (2) quantitative research aimed at assessing physician satisfaction and associated factors; (3) use of validated tools. We performed a narrative synthesis. Results: After screening 8585 records, 368 full text articles were independently checked and finally 24 studies were included for qualitative analysis. The included studies surveyed 20,000 doctors from 12 European countries. The tools and scales used in the analyzed research to measure physician satisfaction varied to a large extent. We extracted all pre-specified factors, reported as statistically significant/non-significant. Analyzed factors were divided into three groups: personal, intrinsic and contextual factors. The majority of factors are modifiable and positively associated with characteristics of contextual factors, such as work-place setting/work environment. In the group of work-place related factors, quality of management/leadership, opportunity for professional development and colleague support have been deemed statistically significant in numerous studies. Conclusions: We identified more studies appraising the effect of contextual factors (such as work environment, work-place characteristics), highlighting a positive association between those factors and physician satisfaction, compared with personal and intrinsic factors. Numerous studies confirmed statistically significant associations between physician satisfaction and quality of management, professional development and colleague support/team climate. Due to the health workforce crisis, knowledge regarding physician satisfaction and associated factors is essential to healthcare managers and policy makers for more stable human resources management.

## 1. Introduction

Physician satisfaction is a critical measure of their wellness [1] which has been negatively associated with physicians’ burnout [1,2]. Satisfaction of physicians is also associated with different healthcare outcomes, including healthcare quality, patient satisfaction and adherence to treatments, and interpersonal aspects of patient care [1,3,4]. However, the factors leading to physician satisfaction need to be further understood [1,5].

Worldwide, many studies have identified determinants of physicians’ job, career, and professional satisfaction. Research has shown that physician satisfaction can be affected by personal and contextual factors, as well as by intrinsic characteristics of the medical profession [1,6,7,8]. Personal factors include age, years of practice, gender, professional achievements, personal satisfaction, nationality, race, and ethnicity [1,7,8]. Contextual factors refer to those in the work environment, including autonomy and work control, workload, job demands, colleague relationships, perceived quality of care, payment and contractual arrangements, academic and administrative duties, income, financial and non-financial incentives, leadership, relationship with support staff, use of electronic health records, and health care reform implementation [1,6,7,8,9]. Intrinsic factors refer to the inherent characteristics of the medical profession, for example patient interactions, demographics and complexity, as well as their own specialty [1]. Still, some personal and contextual factors are understudied and require further attention [7,8,10,11].

Despite the availability of empirical studies evaluating factors associated with physician satisfaction and literature reviews in the field [1,7,8], reports summarizing the determinants of physician satisfaction across Europe are lacking. Literature reviews in this area from other regions, especially North America [7,8], might not capture all the factors influencing European physicians’ satisfaction. Healthcare systems in Europe are not uniform and are constantly transforming, with clear variations among countries. For example, the health care in the United States is based on a non-mandatory and competitive health insurance model in which the interest of stakeholders (e.g., physicians, hospitals, insurers, etc.) is driven by profit. Also, North American physicians have been paid traditionally by fee-for-service schemes and have had significant professional autonomy and independence [12]. In contrast, in Europe, there is quite a diversity of national health systems or social security health care systems. Also, European physicians have been working in these systems with different payment models: salary and capitation being main representative methods [12]. In addition to the income differences, these working environments could change the factors associated with the satisfaction of physicians working in European countries.

To the best of our knowledge, the only research summarizing the level of European physician satisfaction is our own, which found it being moderate [13]. Among European studies differentiating between satisfied and dissatisfied participants, the proportion of satisfied physicians varied from 21% to 95.6% and the weighted percentage of satisfied physicians was 55.3% (95% CI 48.2–62.4) [14]. Furthermore, within the European studies that reported satisfaction as continuous data, the mean pooled levels of satisfaction were: 3.54 (3.29–3.79) in a scale from 1–5, 4.812 (4.70–4.94) in scales from 1–7, 6.12 (5.74–6.52) in a scale from 1–10, and 59.65 (56.80–62.51) in a scale 0–100 [13]. In this article, we aim to synthesize the literature studying the factors associated with the satisfaction of physician working in European hospitals.

## 2. Methods

**Figure 1:**
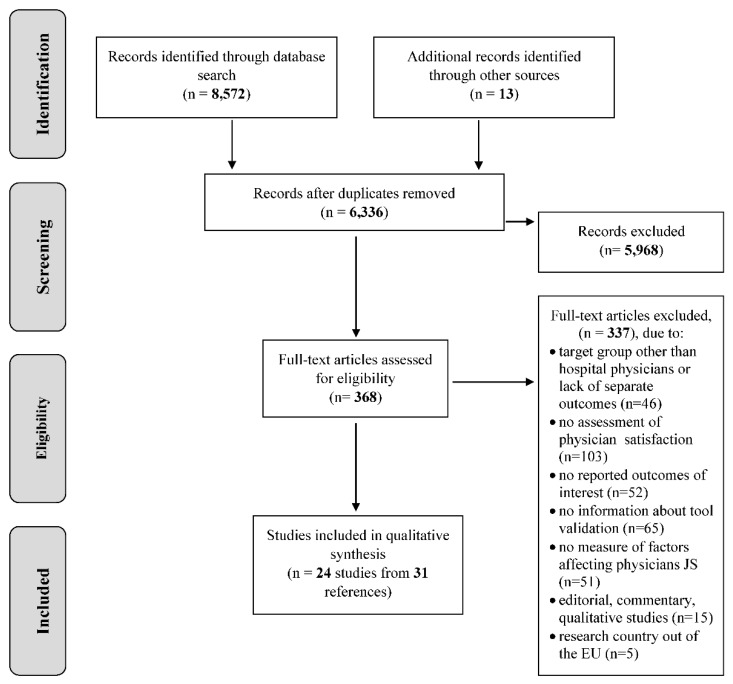
Preferred Reporting Items for Systematic Reviews and Meta-Analyses (PRISMA) flow diagram.

The research protocol of our review was registered in the International prospective register of systematic reviews—PROSPERO (No: 2016 CRD42016053579) [14]. The review process is reported according to the Preferred Reporting Items for Systematic Reviews and Meta-Analyses (PRISMA, Figure 1).

### 2.1. Eligibility Criteria

Study eligibility criteria for our review included: target population: physicians working in EU hospitals, quantitative research aimed at assessing physician satisfaction and associated factors and validated tools used to measure physician satisfaction. Studies focusing only on primary care physicians or other physicians employed only in out-patient clinics, long term care facilities or hospices, as well as solely qualitative studies were excluded.

For studies focusing on hospital healthcare staff, publications were included only if the final results were reported separately for physicians or if they constituted over 50% of the sample size. The same criteria were used for studies concerning the general population of doctors: we included only those studies in which hospital physicians constituted at least 50%. We only included research which used validated tools to measure physician satisfaction. The validity of an instrument is a property of the inference with different degrees of validity of interpretation [15]. Research used a “validated” questionnaire if there was evidence to assess psychometric characteristics of the instrument, including, but not limited to: construct, translational, criterion, face, content, concurrent, predictive, convergent, or discriminant validity, as well as its reliability, internal consistency and temporal stability. In qualitative synthesis we included only those studies which provided information about the validation process or, at least, indicated a reference to the original publication in which the questionnaire was developed and validated. 

### 2.2. Search Methods and Study Identification

We searched five electronic databases: MEDLINE, Embase, PsycINFO, CINAHL and the Cochrane Library for articles published between January 2000 and January 2017. Moreover, DART-EuropeE-theses, portals and websites of professional physician organisations, reference lists of identified reviews and google scholar were searched. No language restrictions were imposed. Our search strategy included both MESH/Emtree terms and free text words including a combination of the following: physician, job satisfaction, work satisfaction, career satisfaction, dissatisfaction, well-being. The complete search strategy for MEDLINE database is presented as Supplementary Materials file S1.

### 2.3. Data Extraction and Quality Assessment

Search results were downloaded to reference management software (EndNote) to remove duplications. In the next step, two reviewers independently screened all titles and abstracts, applying the inclusion/exclusion criteria. Disagreements were resolved through discussion. When consensus was impossible, a third author reviewed the abstract. Full texts of relevant studies were then retrieved and assessed independently by the two reviewers against inclusion/exclusion criteria. When opinions differed, a third reviewer reviewed the article. A dedicated extraction form was designed for data extraction and quality assessment. The data were extracted by one reviewer and checked by another. Two independent reviewers assessed the methodological quality of each study using the 12-item Critical Appraisal of a Survey checklist developed by the Centre for Evidence-Based Management [16]. Because one question was not relevant for our review, 11 items were used for quality assessment of the included studies. Consequent disagreements were resolved by discussion. When no consensus was reached, a third author reviewed the article.

### 2.4. Data Synthesis and Analysis

We extracted the relevant information from the included studies and performed a narrative data synthesis. We collected the following data: country of the research, study settings, study objectives, methodology (study design, participant recruitment, sample size and response rate), characteristics of the sample population (inclusion/exclusion criteria, participant flow, age, gender, years of experience, specialty), working conditions (working hours: part time, full time, payment methods, etc.), tools to measure physician satisfaction (i.e., questionnaires, validation of the instrument, scale of satisfaction, etc.), factors for which association with satisfaction was measured and research results. The factors to be extracted were pre-specified and categorized. We extracted all pre-specified factors, reported as significant and non-significant. All data were extracted by one author and double checked by the second. In data extraction from articles published in languages other than English, authors were supported by native speakers who ensured proper translation quality. 

We intended to perform a meta-analysis to pool the results of factors associated with physician satisfaction. However, meta-analyses are credible and should only be undertaken if the heterogeneity between studies is limited. In this review, the differences between methods for satisfaction measurement and methods for examining associations presenting them were large, so we decided not to perform a meta-analysis. The findings are presented descriptively as a narrative review, and the factors affecting physician satisfaction are categorized in three groups: personal, work-related and other factors. This classification was developed based on literature review [1,6,7] and results of our research.

## 3. Results

### 3.1. Search Results

The electronic databases’ searches yielded 8572 records. Additional 13 articles were identified by hand searching of other resources. After duplicates were removed, titles and abstracts of 6336 records were screened, which resulted in 368 potentially eligible articles. Full texts of these articles were reviewed by two reviewers independently and any disagreements were resolved by discussion. Finally, 24 studies (published in 31 papers) were included in the analysis (Table 1). 337 records were excluded with reasons provided: lack of focus on our target population or no separate results for hospital physicians (46), physician satisfaction not assessed (103), outcomes of interest not reported (52), no information about questionnaire tool validation (65), no measure of factors affecting physician satisfaction (51), non-quantitative study (15) or non-EU research (5). Data from eligible 24 studies were extracted, the quality of the publications was assessed qualitative analysis of the results was undertaken. The study flow is presented on a PRISMA diagram (Figure 1). The complete list of included studies is presented in the Supplementary Materials file S2.

### 3.2. Study Quality

The included studies’ quality ranged between 5 and 10 on the Critical Appraisal evaluation developed by the Centre for Evidence-Based Management, and the mean quality was 7.5. Quality assessment results are presented in the Supplementary Materials file S3. We recognized that the methodological quality of the included studies was a critical factor. Out of the 11 quality criteria, only one study met ten [24], two studies met nine [19,34], eight studies met eight [18,20,23,26,30,32,39,40], 10 studies met seven [21,22,26,27,28,31,33,36,37,38], two studies met six [29,35] and one study met five [17] of them. The criteria which were most rarely met or not reported (in more than half of the analyzed studies) were ‘sample size calculations’ and ‘confidence intervals provided for the main results’.

Six studies had a high response rate (over 75%) [21,31,34,36,38,39], 13 studies had a response rate between 50–75% [17,19,20,22,24,25,27,28,29,32,35,37,40] and in five studies it was lower than 50% [18,23,26,30,33] (Table 1).

### 3.3. Study Characteristic

The included studies enrolled a total of 20,013 physicians from 12 European countries (Table 1). Eight studies were conducted in Germany [18,23,26,27,28,29,32,35], three in the Netherlands [19,36,40], two in Sweden [24,37], two in Finland [17,22], two in Poland [21,39] and one in each of: the United Kingdom [20], Hungary [38], Greece [34], Italy [30], France [31], Austria [25] and Spain [33]. Twenty-two studies were cross-sectional and three were cohort studies [22,36,37]. Since the cohort studies included more than one measurement time point, for our analysis we used baseline data. Sample size varied between 54 and 7090 participants. The included studies were conducted between 1998 and 2013.

The studies included either single specialty (7 studies), multiple specialties or did not provide this information. The most prevalent single specialty within included studied was anesthesiology [19,21,25,31] and surgery [28,29], which were also included among other specialties in studies including physicians of several specialties.

Twenty-two studies provided information on demographic characteristics of the population, while two did not [17,36]. Mean age varied from 29.5 [39] to 49.6 [20]. The proportion of females was between 18% [40] and 62% [17].

The tools used to measure physician satisfaction in the included research varied greatly. In four studies, authors used the Copenhagen Psychosocial Questionnaire [27,28,29,37] and in two studies, the Warr–Cook–Wall Job Satisfaction Scale [20,35]. In other studies, questionnaires developed and validated within previous studies were used (e.g., Minnesota Job Satisfaction Questionnaire; [31] Bovier Questionnaire; [21] Brayfield and Rothe General Index of Job Satisfaction [34]. In 5 studies, researchers used their own questionnaire developed and dedicated to measuring physician satisfaction [23,32,36,38,39].

In the majority of studies, information concerning funding was not reported (13 studies). In eight studies the source of funding was public and two studies reported private funding. One study had mixed funding (public and private).

Given the diversity of analyzed factors explored in the included studies, we categorized them into personal, intrinsic, and contextual factors (Table 2 and Table 3). The detailed results for all analyzed factors are presented in Supplementary Materials file S4.

#### 3.3.1. Personal Factors

##### Physician Age

Of the nine studies that evaluated association between age and physician satisfaction only two studies showed positive association between age and higher levels of job satisfaction [23,35]. These studies reported increase of job satisfaction with age. Moreover, findings from one study show that statistically significant correlation between satisfaction and age is only present in the youngest group of physicians (below 36) [30]. Other studies, in which the relationship between physician satisfaction and age was measured, did not confirm statistically significant association [18,20,28,31,32,34].

##### Gender

Of the 13 included studies that evaluated gender, four observed a statistically significant relationship: male respondents seemed more often satisfied than their female colleagues [17,18,31,36]. In nine studies, no statistically significant association was identified [20,21,22,27,28,32,34,35,36]. Study by Schmit, Jongbloed et al. [36] found a correlation between gender and years in practice: male physicians 20 years in practice were less satisfied than female physicians 20 years in practice as well as their male and female colleagues 10 years in practice. This was specifically related to the administrative aspect of their job satisfaction [36].

##### Years of Experience/Years of Practice

Five studies measured the association between years of experience and physician satisfaction. On the basis of statistically significant associations, the level of satisfaction decreases with the number of years of experience [27,30]. On the other hand, Michiniov et al. reported that long-standing team members reported greater job satisfaction [31]. In two other studies, no significant relationship was identified [21,36].

##### Marital Status or Having a Partner

Relationships between family and job satisfaction were measured in two of the included studies. French et al. examined the influence of being partnered [20] and Gaszynska et al. [21] analyzed the influence of marital status. Neither study found statistically significant associations.

##### Work-Family Conflict (WFC)

Two studies assessed the relationship between WFC and physician satisfaction. Both studies identified a statistically significant impact [22,38] Szilva et al. [38] identified that female physicians reported significantly higher level of WFC than male physicians. Moreover, more female physicians experienced WFC often or extremely often (56% vs. 41%, respectively). Significantly fewer women reported high levels of job satisfaction (55% vs. 66% respectively). Also significantly more female physicians (13%) experienced high levels of job dissatisfaction compared to men (6%). Results of linear regression analyses showed that WFC predicts job dissatisfaction among female, as well as all physicians.

##### Health Status and Life Satisfaction

Three studies examined the relationship between health status and job satisfaction and found statistically significant associations. According to French et al. [20] there was a positive association between excellent health and physician satisfaction. Laubach and Fischbeck [26] found that personal health determined job satisfaction, more so for female physicians. Heponiemi et al. [22] also reported higher job satisfaction for physicians who assessed their health status as excellent and did not report sleeping problems than in employees experiencing sleeping problems.

Rosta et al. [35] identified that general life satisfaction was positively associated with physician satisfaction.

##### Coping Strategies/Psychological Construct

Coping strategies (psychological construct) were explored in five of the included studies (expressed as 16 factors), all of them finding statistically significant relationships [17,25,34,39,40]. The fewer mechanisms are used (coping strategies), the higher the level of job satisfaction [34]. The more often an employee self-motivates himself to a better job performance and approaches the job optimistically, the greater the job satisfaction.

##### Being a Foreign/Internationally Trained Doctor

The association between job satisfaction and being a foreign physician was explored in only one of the included studies [33]. It showed that being a foreign physician was associated with greater career satisfaction when physicians trained outside of Spain in comparison to locally trained physicians.

#### 3.3.2. Intrinsic Factors

##### Specialty

Four of the included studies explored association of medical specialty with physician satisfaction [17,20,22,26]. Due to the heterogeneity of the analyzed specialties it is difficult to interpret the findings. French and colleagues reported that specialty is a significant determinant of job satisfaction [20]. Staff in laboratory medicine, radiology and pediatrics were likely to be more satisfied with their work in comparison to other specialties [20]. In another study psychiatrists were significantly less satisfied with their work compared to others [17]. Moreover, being a specialist is associated with higher satisfaction compared with non-specialists. Heponiemi and colleagues reported that specialists and physicians undergoing specialization training had greater job satisfaction and organizational commitment than non-specialists [22].

##### Patient Interaction

Two of the included studies examined the relationship between patient contact and satisfaction and physician satisfaction. According to Mache and colleagues [28]—with an increase in patients’ overall satisfaction with treatment, physician satisfaction increases. Janus et al. [23] reported that increase in frequency of direct contact between doctor and patient, results in improvement of job satisfaction.

##### Work Engagement

Mache et al. [29] found that job resources have a greater impact on surgeons’ work engagement than their job demands. Moreover, significant correlations between surgeons’ work engagement, job satisfaction and quality of life were found; work engagement mediated the relationship between institutional factors and surgeons’ satisfaction. The authors concluded and suggested that improving physician work engagement could contribute to a more sustainable workplace, in terms of both hospital and individual performance.

#### 3.3.3. Contextual Factors: Workplace Related

##### Hospital Type and Structure

According to one study, working in private, rather than public, hospitals is associated with a higher level of physician satisfaction. Heponiemi et al. [22] reported that physicians working in private hospitals were more satisfied, committed to their job and had less psychological distress and sleeping problems than physicians working in public hospitals. Physicians working in the private sector experienced better organizational justice and job control.

##### Management/Leadership

In six studies the impact of management and leadership quality on physician satisfaction was assessed and their important role was observed [19,22,26,27,37,40]. Beuzekom et al. showed that job satisfaction was most strongly related to planning/coordination and hierarchy. Poor planning/coordination had the most negative effect on anesthetists’ job satisfaction [19]. The same research shows that job satisfaction was most strongly related to maintenance, access to information, teamwork and hierarchy. All of these factors depend on management quality [19]. Stromgren et al. assessed “trust regarding management” [37]. Half (51%, 114) of the physicians reported high trust regarding management. Physicians rated the highest level of summed social capital, especially with regard to mutual trust between employees. The authors reported that increased social capital predicted increased job satisfaction.

Heponiemi and colleagues [22] assessed organizational justice using a scale consisting of four subscales: procedural, interpersonal, informational and distributive justice. Findings showed that better organizational justice in the private sector could partly explain more positive attitudes and better well-being in physicians working there. According to the authors, to improve physician satisfaction, management in public healthcare organizations should better consider physicians’ working conditions [22]. Laubach and Fischbeck [26] reported that among the explanatory variables in their study “superiors and hierarchy” showed the highest beta-weight and proved the most significant predictors for physician satisfaction with “work and profession”. Moreover, significant associations between gender and age were found in the categories of job satisfaction and “superiors and hierarchy”: the values of female doctors from the “work and profession” scale decreased with age. The oldest females, however, had the highest values. Male physicians’ results varied by age, but the oldest group scored lowest. According to the authors, the significant association between gender and age in the scales “superiors and hierarchy” can be interpreted with differences in professional status and functions [26].

Visser et al. reported that the feeling of being poorly managed and resourced was associated with diminished physician satisfaction [40]. The study by Mache et al. [27] found that leadership quality was significantly positively associated with physician satisfaction. Scores on leadership quality and amount of regular feedback on work performance indicate that physicians at private non-profit hospitals rated their supervisors and colleagues higher than physicians working at other hospitals.

Jonsson aimed to analyze similarities and differences in physicians’ experience of quantitative and qualitative demands, control, role conflicts, role clarity, social support and job satisfaction in 2002 and 2009 [24]. The results showed that some aspects of the psychosocial work environment improved between 2002 and 2009 and that variables predicting job satisfaction were quite stable over time. Job satisfaction, role clarity and social support were experienced as more positive in 2009 compared to 2002 [24].

Mascia et al. [30] contributes to the understanding of hospital restructuring through the adoption of clinical directorate models by exploring how structural characteristics of organizational arrangements influence physicians’ overall job satisfaction. Their findings demonstrated how adoption of “process integration” directorates is positively associated with job satisfaction, suggesting that physicians may be more accepting of process-integration departments compared to specialty-integration departments. They identified that the type of organizational arrangement adopted within hospitals is significantly associated with improved physician job satisfaction. Specifically, “Hospital trust” and “Research hospital” were positively associated with job satisfaction, whereas the variable “Local health authority hospital” was negatively related to perceived increase in physician satisfaction. In the same study, the effects of hospital restructuring on overall physician job satisfaction were examined, finding that physicians with high openness to experience scores were more receptive to the positive impacts of change on overall job satisfaction.

##### Opportunity for Professional Development

Five studies examined relationship between opportunities for professional development and physician satisfaction and found that when physicians have opportunities for professional development and trainings, are intellectually stimulated at work and receive feedback about the work they do, their satisfaction increases significantly [23,27,28,29,40]. One study found no statistically significant relationship between research/teaching activities or international exchange and satisfaction [23].

##### Colleague Support (Team Work, Team Relations)

There is substantial evidence on the relationship between colleague support and physician satisfaction. Among the 24 included studies, 12 assessed this correlation (expressed in several different ways using 31 factors). Findings from 11 studies (expressed using 23 factors) show that greater recognition, positive team climate and support from colleagues is associated with a significantly greater job satisfaction [17,23,24,25,26,27,28,29,31,37,40]. Interesting data were reported by Michinov and colleagues, [31] who stated that for physicians working in smaller teams, job satisfaction is higher.

##### Access to Resources

Findings from two studies provided some evidence of higher physician satisfaction with access to material resources [19] or specialized technologies [23].

#### 3.3.4. Contextual Factors: Job Related Factors

##### Workload and Job Demand

Three analyzed studies show significant negative association between both psychological stress at work, amount of work and physician satisfaction [17,22,40].

##### Work Control

Four studies examined relationship between work control and job satisfaction and in three of them, significant relationships were observed [20,27,28]. Equal treatment of employees was associated with higher job satisfaction in one study and in two studies there was significant positive correlation between degree of freedom at work/influence on work and satisfaction. Findings of another study and regression analyses of the two aforementioned studies did not confirm statistically significant association between influence at work/degree of freedom at work and physician satisfaction [29].

##### Work Stability

Two studies examined and found significant associations between work stability and job satisfaction. According to Visser et al. [40] better job security was associated with higher job satisfaction. Heponiemi et al. reported that part-time employment was associated with lower job satisfaction [22].

##### Being a Chief

Three studies examined the relationship between being a chief and job satisfaction. Based on the results of two included studies, [18,35] it can be said that being a chief doctor is associated with significantly higher levels of job satisfaction. In one study the relationship between being a chief and physician satisfaction was not deemed statistically significant [30].

##### Income and Non-Financial Incentives

Only one study examined the relationship between level of remuneration and physician satisfaction [20]. Higher income was not associated with job satisfaction for associate specialists. The only important factor related to the level of satisfaction was the level of earnings in the range of 35 k–50 k pounds. In two studies, non-financial incentives were explored and both showed statistically significant association for physician satisfaction: having received non-monetary incentives during the last year [33] and feeling valued [40]. On the basis of these two studies, after providing incentives and a sense of appreciation to employees, satisfaction significantly increased.

#### 3.3.5. Contextual Factors: Others Factors

##### Intention to Leave

Beuzekom with colleagues [19] investigated the extent to which latent risk factors are related to job satisfaction and intention to leave for specialist anesthetists and trainee anesthetists. They found that for anesthetists, job satisfaction was moderately to highly correlated with intention to leave. For specialist anesthetists, latent risk factors accounted for a significant proportion of variance in job satisfaction and intention to leave. For trainee anesthetists, latent risk factors accounted for a significant proportion of variance in job satisfaction, but not in intention to leave.

##### Prior Achievements

Schmit Jongbloed et al. [36] investigated the relationship between physicians’ prior achievements (before, during and after medical school) and job satisfaction, and tested two lines of reasoning that prior achievements influence job satisfaction positively or negatively, respectively. Curriculum types (problem-based learning versus traditional) were examined, but according to the findings, did not significantly influence job satisfaction.

## 4. Discussion

This study is the first systematic review summarizing factors associated with European hospital physician satisfaction. We identified numerous studies evaluating factors significantly associated with physician satisfaction, including personal, intrinsic and contextual factors, with more research appraising the effect of work-place characteristics and work environment factors than personal and intrinsic factors. In general, our findings are aligned with other reviews on factors affecting physician satisfaction [6,7,41,42]. Results from studies evaluating factors associated with the satisfaction of physicians in Europe are in line with those describing these factors described in other countries and regions. Nevertheless, studies in the field in Europe also contribute to areas where there was a lack of evidence.

Regarding personal factors associated with physician satisfaction, numerous studies have largely studied age and gender factors within Europe (Table 3). Despite several studies reporting controversial or inconclusive results regarding the association of physician satisfaction and age or years of experience [21,22,27,30,35,36], there is evidence that older and experienced physicians tend to be more satisfied than their younger counterparts [22,31,35]. Additionally, several studies in Europe have reported that male physicians appear to be more satisfied than their female colleagues [17,18,31,36]. We want to highlight the correlation between gender and years of practice identified in a longitudinal study conducted in the Netherlands. Authors of this study stated that male physicians being two decades in practice were less satisfied than female counterparts. The same applied to their male and female colleagues being a decade in practice [36].

Furthermore, physicians reporting good health and life satisfaction were more likely to be satisfied with their jobs [20,26,35], particularly women [22], and internationally trained physicians are more satisfied than those trained locally [33] The presence of work-family conflict [22,38], especially among women [38], use of coping strategies [17,25,34,39,40] and being a foreign trained doctor [33] were other important factors associated with physician satisfaction studied in different EU countries.

Regarding contextual factors associated with European hospital physician satisfaction, we identified that physicians were more satisfied when working in private institutions [22], trusted and well-planned organizations with good management and leadership strategies [19,22,26,27,37,40] and organizations with integrated research processes [30]. Additionally, better levels of satisfaction were reported among physicians who had professional autonomy [20,27,28], work stability [22,40], access to technology and resources to provide care [19,23,29], a leadership role [18,35], opportunities for professional development [23,27,28,29,40], peer support [17,23,24,25,26,27,28,29,31,37,40], received non-financial incentives [33,40], and worked in small teams [31]. Three European studies identified that physician satisfaction could deteriorate under heavy workload [17,22,40].

Concerning intrinsic factors influencing physician satisfaction, we identified European studies exploring the association between medical specialty and physician satisfaction [17,20,22,26]. These studies reported better levels of satisfaction among physicians of laboratory medicine, radiology, and pediatrics [20], and poorer among general doctors or trainees [22] and psychiatrists [17]. Additionally, other studies identified that physician satisfaction is associated with patient satisfaction and frequent patient interactions [23,28].

### 4.1. Limitations

Our systematic review has some limitations that need to be acknowledged. Included studies were conducted in many European countries, with different healthcare systems. Due to differences between health systems and physicians’ working conditions, satisfaction and influence of analyzed factors varied. Thus, the methodological variability of the included studies is an important issue, making it difficult to compare and standardize the results. Regarding methodological quality assessment, in about half of the analyzed studies, the reporting was incomplete. In many studies information regarding the representativeness of the target group, sample size calculations, confidence intervals and other statistical parameters characterizing the main results was lacking. Future studies of factors affecting physician satisfaction should focus on the quality of reporting as this is a critical issue.

Another very important limitation was the lack of information on validation of the questionnaires used to measure job satisfaction. This limited the inclusion of possibly relevant studies. In addition, the scales used to measure job satisfaction differed significantly and studies did not report information on variability. In both of these situation we were unable to include these studies.

The number of studies included for analysis of particular factors differed significantly, e.g., the impact of income on physician satisfaction was analyzed in few studies (although a variety of studies confirmed that higher income is associated with greater professional satisfaction), while many research investigated others factors (e.g., hospital structure, relationship with co-workers, professional development opportunities). This may have partially resulted from excluding many studies for lack of validation information.

Another fact that needs to be acknowledged is that we focused on research conducted in EU countries, without analyzing research outside the EU.

### 4.2. Implications

Friedberg and colleagues reported that physician dissatisfaction might suggest that medical doctors or healthcare units where doctors work are providing health services of insufficient quality [6]. Multiple studies confirm influence of burnout syndrome on physician satisfaction [1,2,6]. According to Kravitz, if physician dissatisfaction affects quality of health services, then physician dissatisfaction is a public health issue [43]. Some evidence regarding the relationship between physician satisfaction and patient satisfaction suggests that if physicians are satisfied, their patients are also more satisfied, but the data is limited. Physician satisfaction has a positive impact on patients’ compliance and actions in managing chronic diseases [1]. Dissatisfied physicians tend to have riskier prescribing profiles, less adherent patients, less satisfied patients, and their healthcare quality might possible decrease [3].

Physician satisfaction and physician well-being are not synonyms, but the same factors affect both, impacting physician behavior and consequently, the quality of medical care [23]. Considering the European physician workforce crisis [44,45], studies that provide evidence on the relationship between physician satisfaction and problems such as workload, WFC and intention to leave, would provide crucial information on this subject. According to the results of our literature review, most factors affecting physician satisfaction are modifiable and closely connected to the workplace. Physician satisfaction needs to be measured and monitored to improve working conditions and increase employment stability in the healthcare sector.

## 5. Conclusions

This is the first study summarizing the satisfaction determinants of physicians working in European hospitals. Numerous European studies have evaluated the association of physician satisfaction with age and gender, with the results being inconclusive. Regarding contextual factors, there are studies highlighting the positive association between physician satisfaction and certain environments (i.e., private institutions, organizations with good management and leadership strategies, and those with integrated research processes) and working conditions (i.e., professional autonomy, work stability, access to technology and resources, and opportunities for professional development). It is important to highlight how good relationships with colleagues, non-financial incentives, workload, and number of co-workers could affect physician satisfaction. Similarly, specialty, patient interaction, health status, life satisfaction, WFC, use of coping strategies, and intention to leave are other relevant factors that have been studied among different European countries.

Future studies evaluating factors associated with the satisfaction of physicians should continue evaluating external factors, workplace and job related ones, with longitudinal study designs. Additionally, these studies should undertake in-depth analyses of personal and intrinsic factors associated with the satisfaction of medical practitioners and control their results with these factors when studying contextual ones.

## Figures and Tables

**Table 1:** Characteristics of the studies included in the analysis.

No	Study ID	Country	Period Years	Target Group/Medical Specialization	Response Rate	Number of Physicians Included in the Analysis	Tools used to Measure Physician Satisfaction
1	Aalto et al. (2014) [17]	Finland	2010	Finnish physicians	55%	1916	Job Diagnostic Survey (JDS)
2	Bauer and Gronenberg (2013) [18]	Germany	2012–2013	Physicians in German hospitals	18%	7090	Job Diagnostic Survey (JDS)
3	van Beuzekom et al. (2013) [19]	Netherlands	NR	Anaesthetists, trainee anaesthetists and nurse anaesthetists from three university hospitals	67% 56%	109 specialists and 46 trainees	Leiden Quality of Work Questionnaire (scale for JS)
4	French et al. (2007) [20]	United Kingdom	2001	Staff and associate specialists (SAS doctors) working in NHS Scotland	50% 52%	251 staff grades and 100 for associate specialists	Warr—Cook—Wall Job Satisfaction Scale
5	Gaszynska et al. (2014) [21]	Poland	2013	Senior specialist anaesthetists working within the Lodzkie Voivodship	77%	136	Bovier et al. questionnaire
6	Heponiemi et al. (2008–2015) [22]	Finland	2006	Finnish physicians	57%	2652	Job Diagnostic Survey (JDS)
7	Janus et al. (2008) [23]	Germany	2004–2005	Physicians at the university hospital in Hannover	46.5%	390	Authors’ own questionnaire
8	Jönsson (2012) [24]	Sweden	2002–2009	Physicians and nurses registered in a county council in the south of Sweden (Region Skane)	65% 45%	In 2002: 499 physicians In 2009: 359 physicians	Questionnaire based on General Nordic Questionnaire (QPS Nordic) for Psychological and Social Factors at Work
9	Kinz et al. (2005) [25]	Austria	2003	Anaesthetists from the University Department of Anaesthesiology and Critical Care Medicine, Innsbruck	66%	89	Olson and Stewart Global Job Satisfaction Scale
10	Laubach and Fischbeck (2007) [26]	Germany	2001–2002	Physicians at the university hospital of Mainz	42%	438	General and specific satisfaction in life developed by Fahrenberg et al.
11	Mache et al. (2009) [27]	Germany	2008	German hospital physicians	67%	203	Copenhagen Psychological Questionnaire (COPSOQ)
12	Mache et al. (2012) [28]	Germany	2009–2010	Patients and surgeons (full-time employed junior doctors or residents specializing in surgery) working in surgery hospital departments in Germany	65%	98	Copenhagen Psychological Questionnaire (COPSOQ)
13	Mache et al. (2014) [29]	Germany	2009–2011	Surgeons in hospital departments for surgery in German hospitals	63%	123	Copenhagen Psychological Questionnaire (COPSOQ)
14	Mascia et al. (2014) [30]	Italy	2006– 2009	Physicians in 18 clinical directorates employed by ten hospitals in the I-NHS	27%	336	Overall Job Satisfaction Scale
15	Michinov et al. (2008) [31]	France	2006–2006	Physician anaesthesiologists and nurse anaesthetists working in an anaesthesia team	78%	74	Minnesota Job Satisfaction Questionnaire
16	Ommen et al. (2009) [32]	Germany	2002	Physicians working in 4 different German hospitals	61%	277	Authors’ own questionnaire
17	Peña-Sánchez et al. (2014) [33]	Spain	2009–2010	Specialist physicians working in hospitals of Andalusia, Spain	41%	121	4CornerSat Questionnaire developed by Lepnurm
18	Psilopanagioti et al. (2012) [34]	Greece	2011	Physicians working at the University Hospital of Patras	87%	130	Brayfield and Rothe General Index of Job Satisfaction
19	Rosta and Gerber (2008) [35]	Germany	2006	Physicians in German hospitals	58%	1890	Warr—Cook—Wall Job Satisfaction Scale
20	Schmit Jongbloed et al. (2014) [36]	Netherlands	2009–2010	Graduates who started medical training at the University of Groningen in 1982, 1983, 1992 and 1993.	88%	523	Authors’ own questionnaire
21	Strömgren et al. (2016) [37]	Sweden	2012	Physicians in intensive care, emergency, surgical and medical units in 5 hospitals	53%	224	Copenhagen Psychological Questionnaire (COPSOQ)
22	Szilvia et al. (2009) [38]	Hungary	2005–2007	Female Hungarian physicians (male physicians as control group)	76%	440	Authors’ own questionnaire
23	Tartas et al. (2011) [39]	Poland	Part I: 1999–2005 Part II: 2008–2009	Medical doctors who graduated from The Medical University of Gdansk	different in subgroup: 87% and 21%	54	Authors’ own questionnaire
24	Visser et al. (2003) [40]	Netherlands	1998	Dutch medical specialists	66%	1435	Consultants’ Mental Health Questionnaire

**Table 2:** Factors affecting physician satisfaction (analyzed in studies included in the systematic review).

Personal Factors	Intrinsic Factors	Contextual Factors		
		Workplace Related	Job Related	Other
Age [18,20,22,28,30,31,32,34,35] Gender [17,18,20,21,22,27,28,31,32,34,35,36] Years of experience/practice [21,27,30,31,36] Marital status or having a partner [20,21] Work-family conflict [22,38] Health status [20,22,26] Life satisfaction [35] Coping strategies/psychological construct [17,25,34,39,40] Being a foreign/internationally trained doctor [33]	Specialty [17,20,22,26] Patients interactions [23,28] Work engagement [29]	Hospital type and structure [22] Management and leadership [19,22,26,27,37,40] Opportunity for professional development [23,27,28,29,40] Colleague support [17,23,24,25,26,27,28,29,31,37,40] Access to resources [19,23]	Workload and job demands [17,22,40] Work control [27,28,29,30] Work stability [22,40] Being a chief [18,30,35] Income and non-financial incentives [20,33,40]	Intention to leave [19] Prior achievement [36]

**Table 3:** Factors statistically and non-statistically significant for physician satisfaction (analyzed in studies included in the systematic review).

Factors		Statistically Significant	Statistically Non-Significant
**Personal factors**	Age	Heponiemi et al. [22] Mascia et al. [30] *(under 36)* Rosta and Gerber [35]	Bauer and Gronenberg [18] Mache et al. [27] Mascia et al. [30] *(for other age groups than under 36)* Ommen et al. [32] Michniov et al. [31] Psilopanagioti et al. [34]
	Gender	Aalto et al. [17] *(female)* Bauer and Gronenberg [18] *(female)* Michinov et al. [31] *(female)* Schmit Jongbloed [36]	Bauer and Gronenberg [18] *(male)* French et al. [20] Gaszynska et al. [21] Heponiemi et al. [22] Mache et al. [27] Mascia et al. [30] Ommen et al. [32] Psilopanagioti et al. [34] Rosta and Gerber [35]
	Years of experience/practice	Mache et al. [28] Mascia et al. [30] Michinov et al. [31]	Gaszynska et al. [21] Schmit Jongbloed et al^.^ [36]
	Marital status or having a partner		French et al. [20] Gaszynska et al. [21]
	Work-family conflict	Heponiemi et al. [22] Szilvia et al. [38]	
	Health status	French et al. [20] *(staff)* Heponiemi et al. [22] Laubachand Fischbeck [26]	French et al. [20] *(associated specialists)*
	Life satisfaction	Rosta and Gerber [35]	
	Coping strategies/psychological construct	Aalto et al. [17] Kinz et al. [25] Psilopanagioti et al. [34] Tartas et al. [39] Visser et al. [40]	
	Being a foreign doctor	Peña-Sánchez et al. [33]	
**Intrinsic factors**	Specialty	Aalto et al. [17] French et al. [20] Heponiemi et al. [22]	
	Patients interactions	Janus et al. [23] Mache et al. [28]	
	Work engagement	Mache et al. [29]	
**Contextual factors**	Workplace Related		
	Hospital type and structure	Heponiemi et al. [22]	
	Management and leadership	van Beuzekom et al. [19] Heponiemi et al. [22] Jönsson [25] Mache et al. [27] Mascia et al. [30] *(research, trust and LHA hospitals)* Strömgren et al. [37] Visser et al. [40]	Mascia et al. [30] *(teaching hospitals)*
	Opportunity for professional development	Janus et al. [23] Mache et al. [27] Mache et al. [28] Mache et al. [29] Visser et al. [40]	Janus et al. [23]
	Colleague support	Aalto et al. [17] Janus et al. [23] Jönsson [24] Kinz et al. [25] Michinov et al. [31] Strömgren et al. [37] Mache et al. [29] Visser et al. [40]	Ommen et al. [32]
	Access to resources	van Beuzekom et al. [19] *(anesthestists)* Janus et al. [23]	van Beuzekom et al. [19] *(trainee anesthetists)*
	Job related		
	Workload and job demands	Aalto et al. [17] Heponiemi et al. [22] Visser et al. [40]	
	Work control	French et al. [20] Mache e al. [28] *(subgroups)*	Mache [28] *(subgroups)*
	Work stability	Heponiemi et al. [22] Visser et al. [40]	
	Being a chief	Bauer and Gronenber [18] Rosta and Gerber [35]	Mascia et al. [30]
	Income and non-financial incentives	French et al. [20] *(in one range of income per year)* Peña-Sánchez et al. [33] Visser et al. [40]	French et al. [20] *(for other income range)*
	Other		
	Intention to leave	van Beuzekom et al. [19] *anesthetists*	
	Prior achievement		Schmit Jongbloed et al. [36]

## Supplementary Materials

The following are available online at http://www.mdpi.com/1660-4601/15/11/2546/s1. All the data and information included in this review can be found in the Supplementary files.
