# Supplementary material for: Factors Associated with Satisfaction of Hospital Physicians: A Systematic Review on European Data
Source: Int J Environ Res Public Health. 2018 Nov 13;15(11):2546. doi: 10.3390/ijerph15112546 (PMC6266839; doi:10.3390/ijerph15112546)
Supplement: Supplementary file 1 [file ijerph-15-02546-s001.pdf]

SUPPLEMENTARY MATERIALS

Supplementary file - S1

**Database search strategy in MEDLINE via OVID database**

1. (physicians or physicians role).sh.

2. workload.sh.

3. burnout, professional.sh.

4. job satisfaction.sh.

5. 2 or 3 or 4

6. 1 and 5

7. Physicians.af.

8. doctor.af.

9. medic.af.

10. medical practitioner.af.

11. specialist.af.

12. intern.af.

13. 7 or 8 or 9 or 10 or 11 or 12

14. job satisfaction.mp. or work satisfaction.af. [mp=title, abstract, original title, name of substance word, subject heading word, keyword heading word, protocol supplementary concept word, rare disease supplementary concept word, unique identifier]

15. career satisfaction.af.

16. job dissatisfaction.mp. or work dissatisfaction.af. [mp=title, abstract, original title, name

of substance word, subject heading word, keyword heading word, protocol supplementary concept word, rare disease supplementary concept word, unique identifier]

17. career dissatisfaction.af.

18. burnout.af.

19. workload.af.

20. 14 or 15 or 16 or 17 or 18 or 19

21. 13 and 20

22. 6 or 21

23. limit 22 to (humans and yr="2000 -Current")

24. Europe.af.

25. european.af.

26. (Austria or Belgium or Bulgaria or Croatia or Cyprus or Czech Republic or Denmark or Estonia or Finland or France or Germany or Greece or Hungary or Ireland or Italy or Latvia or Lithuania or Luxembourg or Malta or Netherlands or Poland or Portugal or Romania or Slovakia or Slovenia or Spain or Sweden or United Kingdom).af.

27. 24 or 25

28. 26 or 27

29. 23 and 28

**Supplemantary file S2**

## S2 – List of the studies included in the analysis


1. Aalto, A.M.; Heponiemi, T.; Vaananen, A.; Bergbom, B.; Sinervo, T.; Elovainio, M. Is working in culturally diverse working environment associated with physicians' work-related well-being? A cross-sectional survey study among Finnish physicians. *Health Policy* **2014**, *117*, 187–194.
2. Bauer, J.; Groneberg, D.A. Distress among physicians in hospitals—An investigation in Baden-Wurttemberg, Germany. *Deutsche Medizinische Wochenschrift (1946)* **2013**, *138*, 2401–2406.
3. Van Beuzekom, M.; Akerboom, S.; Boer, F.; Dahan, A. Influence of latent risk factors on job satisfaction, job stress and intention to leave in anaesthesia teams: A cross-sectional survey. *Eur. J. Anaesthesiol.* **2013**, *30*, 222–228.
4. French, F.; Ikenwilo, D.; Scott, A. What influences the job satisfaction of staff and associate specialist hospital doctors? *Health Serv. Manag. Res.* **2007**, *20*, 153–161.
5. Gaszynska, E.; Stankiewicz-Rudnicki, M.; Szatko, F.; Wieczorek, A.; Gaszynski, T. Life satisfaction and work-related satisfaction among anesthesiologists in Poland. *Sci. World J.* **2014**, *2014*, 601865.
6. a. Heponiemi, T.; Aalto, A.M.; Puttonen, S.; Vänskä, J.; Elovainio, M. Work-related stress, job resources, and well-being among psychiatrists and other medical specialists in Finland. *Psychiatr. Serv.* **2014**, *65*, 796–801.
   b. Heponiemi, T.; Kouvonen, A.; Vanska, J.; Halila, H.; Sinervo, T.; Kivimaki, M. Effects of active on-call hours on physicians' turnover intentions and well-being. *Scand. J. Work Environ. Health* **2008**, *34*, 356–363.
   c. Heponiemi, T.; Kouvonen, A.; Virtanen, M.; Vanska, J.; Elovainio, M. The prospective effects of workplace violence on physicians' job satisfaction and turnover intentions: The buffering effect of job control. *BMC Health Serv. Res.* **2014**, *14*, 19.
   d. Heponiemi, T.; Kuusio, H.; Sinervo, T.; Elovainio, M. Job attitudes and well-being among public vs. private physicians: Organizational justice and job control as mediators. *Eur. J. Public Health* **2010**, *21*, 520–525.
   e. Heponiemi, T.; Puttonen, S.; Elovainio, M. On-call work and physicians' well-being: Testing the potential mediators. *Occup. Med. Oxf. Engl.* **2014**, *64*, 352–357.
7. Janus, K.; Amelung, V.E.; Baker, L.C.; Gaitanides, M.; Schwartz, F.W.; Rundall, T.G. Job satisfaction and motivation among physicians in academic medical centers: Insights from a cross-national study. *J. Health Politics Policy Law* **2008**, *33*, 1133–1167.
8. Jonsson, S. Psychosocial work environment and prediction of job satisfaction among Swedish registered nurses and physicians—A follow-up study. *Scand. J. Caring Sci.* **2012**, *26*, 236–244.
9. Kinzl, J.F.; Knotzer, H.; Traweger, C.; Lederer, W.; Heidegger, T.; Benzer, A. Influence of working conditions on job satisfaction in anaesthetists. *Br. J. Anaesth.* **2005**, *94*, 211–215.
10. Laubach, W.; Fischbeck, S. Job satisfaction and the work situation of physicians: A survey at a German University hospital. *Int. J. Public Health* **2007**, *52*, 54–59.
11. Mache, S.; Vitzthum, K.; Nienhaus, A.; Klapp, B.F.; Groneberg, D.A. Physicians' working conditions and job satisfaction: Does hospital ownership in Germany make a difference? *BMC Health Serv. Res.* **2009**, *9*, 148.

12. Mache, S.; Vitzthum, K.; Klapp, B.F.; Groneberg, D.A. Improving quality of medical treatment and care: Are surgeons' working conditions and job satisfaction associated to patient satisfaction? *Langenbeck's Arch. Surg./Deutsche Gesellschaft fur Chirurgie* **2012**, *397*, 973–982.
13. Mache, S.; Vitzthum, K.; Klapp, B.F.; Danzer, G. Surgeons' work engagement: Influencing factors and relations to job and life satisfaction. *Surgeon J. R. Coll. Surg. Edinb. Irel.* **2014**, *12*, 181–190.
14. Mascia, D.; Morandi, F.; Cicchetti, A. Hospital restructuring and physician job satisfaction: An empirical study. *Health Policy* **2014**, *114*, 118–127.
15. a. Michinov, E.; Olivier-Chiron, E.; Rusch, E.; Chiron, B. Influence of transactive memory on perceived performance, job satisfaction and identification in anaesthesia teams. *Br. J. Anaesth.* **2008**, *100*, 327–332.
b. Chiron, B.; Michinov, E.; Olivier-Chiron, E.; Laffon, M.; Rusch, E. Job satisfaction, life satisfaction and burnout in French anaesthetists. *J. Health Psychol.* **2010**, *15*, 948–958.
16. Ommen, O.; Driller, E.; Köhler, T.; Kowalski, C.; Ernstmann, N.; Neumann, M.; Steffen, P.; Pfaff, H. The relationship between social capital in hospitals and physician job satisfaction. *BMC Health Serv. Res.* **2009**, *9*, 81.
17. Peña-Sánchez, J.N.; Lepnurm, R.; Morales-Asencio, J.M.; Delgado, A.; Domagała, A.; Górkiewicz, M. Factors identified with higher levels of career satisfaction of physicians in Andalusia, Spain. *Health Psychol. Res.* **2014**, *2*, 58–62.
18. Psilopanagioti, A.; Anagnostopoulos, F.; Mourtou, E.; Niakas, D. Emotional intelligence, emotional labor, and job satisfaction among physicians in Greece. *BMC Health Serv. Res.* **2012**, *12*, 463.
19. Rosta, J.; Nylenna, M.; Aasland, O.G. Job satisfaction among hospital doctors in Norway and Germany. A comparative study on national samples. *Scand. J. Public Health* **2009**, *37*, 503–508.
20. Schmit Jongbloed, L.J.; Schönrock-Adema, J.; Borleffs, J.C.; Stewart, R.E.; Cohen-Schotanus, J. The influence of achievement before, during and after medical school on physician job satisfaction. *Adv. Health Sci. Educ. Theory Pract.* **2014**, *19*, 581–595.
21. Stromgren, M.; Eriksson, A.; Bergman, D.; Dellve, L. Social capital among healthcare professionals: A prospective study of its importance for job satisfaction, work engagement and engagement in clinical improvements. *Int. J. Nurs. Stud.* **2016**, *53*, 116–125.
22. Szilvia, Á.; Zsuzsa, G.; Krisztina, L. High prevalence of job dissatisfaction among female physicians: Work-family confl ict as a potential stressor. *Orvosi Hetilap* **2009**, *150*, 1451–1456.
23. a. Tartas, M.; Walkiewicz, M.; Budzinski, W.; Majkowicz, M.; Wojcikiewicz, K. The sense of coherence and styles of success in the medical career: A longitudinal study. *BMC Med. Educ.* **2014**, *14*, 254.
b. Tartas, M.; Walkiewicz, M.; Majkowicz, M.; Budzinski, W. Psychological factors determining success in a medical career: A 10-year longitudinal study. *Med. Teach.* **2011**, *33*, e163–e172.
c. Walkiewicz, M.; Tartas, M.; Majkowicz, M.; Budzinski, W. Academic achievement, depression and anxiety during medical education predict the styles of success in a medical career: A 10-year longitudinal study. *Med. Teach.* **2012**, *34*, e611–e619.
24. Visser, M.R.M.; Smets, E.M.A.; Oort, F.J.; de Haes, H.C.J. Stress, satisfaction and burnout among Dutch medical specialists. *CMAJ Can. Med. Assoc. J.* **2003**, *168*, 271–275.

**Supplementary file S3**

**Table 1. Results of the studies' quality assessment based on Critical Appraisal of a Survey developed by the Centre for Evidence-Based Management**

| | Study/Question | Did the study address a clearly focused question/ issue? | Is the research method (study design) appropriate for answering the research question? | Is the method of selection of the subjects (employees, teams, divisions, organizations) clearly described? | Could the way the sample was obtained introduce (selection) bias? | Was the sample of subjects representative with regard to the population to which the findings will be | Was the sample size based on pre-study considerations of statistical power? | Was a satisfactory response rate achieved? | Are the measurements (questionnaires) likely to be valid and reliable? | Was the statistical significance assessed? | Are confidence intervals given for the main results? | Could there be confounding factors that haven't been accounted for? | Overall score |
|---|---|---|---|---|---|---|---|---|---|---|---|---|---|
| 1 | Aalto et al. (2014)[17] | Yes | Yes | No | No | No | No | No | Yes | Yes | No | Yes | 5 |
| 2 | Bauer et al. (2016)[18] | Yes | Yes | Yes | Yes | Can'tell | Can'tell | No | Yes | Yes | Yes | Yes | 8 |
| 3 | van Beuzekom et al. (2013)[19] | Yes | Yes | Yes | Yes | Can'tell | Can'tell | Yes | Yes | Yes | Yes | Yes | 9 |
| 4 | French et al. (2007)[20] | Yes | Yes | Yes | Yes | Yes | No | No | Yes | Yes | No | Yes | 8 |
| 5 | Gaszynska et al. (2014)[21] | Yes | Yes | Can'tell | Can'tell | Yes | Can'tell | Yes | Yes | Yes | Yes | No | 7 |

| | | | | | | | | | | | | | |
|---|---|---|---|---|---|---|---|---|---|---|---|---|---|
| 6 | Heponiemi et al. (2008-2015)[22] | Yes | Yes | Yes | Yes | Yes | Can'tell | No | Yes | Yes | No | No | 7 |
| 7 | Janus et al. (2008)[23] | Yes | Yes | Yes | No | Yes | Can'tell | Yes | Yes | Yes | No | Yes | 8 |
| 8 | Jönsson (2012)[24] | Yes | Yes | Yes | Yes | Yes | Yes | Yes | Yes | Yes | No | Yes | 10 |
| 9 | Kinz et al. (2005)[25] | Yes | Yes | Yes | Yes | Can'tell | Yes | No | Can'tell | Yes | Yes | Yes | 8 |
| 10 | Laubach and Fischbeck (2007)[26] | Yes | Yes | No | Yes | Can'tell | No | Yes | Yes | Yes | No | Yes | 7 |
| 11 | Mache et al. (2009)[27] | Yes | Yes | Yes | No | Yes | No | Yes | Yes | Yes | No | No | 7 |
| 12 | Mache et al. (2012)[28] | Yes | Yes | Yes | Yes | No | No | No | Yes | Yes | No | Yes | 7 |
| 13 | Mache et al. (2014)[29] | Yes | Yes | No | Can'tell | Can'tell | Yes | Can'tell | Yes | Yes | No | Yes | 6 |
| 14 | Mascia et al. (2014)[30] | Yes | Yes | No | Yes | Yes | Can'tell | Yes | Yes | Yes | No | Yes | 8 |
| 15 | Michinov et al. (2008)[31] | Yes | Yes | Yes | Can'tell | Yes | Can'tell | Can'tell | Yes | Yes | Yes | No | 7 |
| 16 | Ommen et al. (2009)[32] | Yes | Yes | Yes | Yes | Can'tell | Can'tell | No | Yes | Yes | Yes | Yes | 8 |
| 17 | Peña-Sánchez et al. (2014)[33] | Yes | Yes | Yes | Yes | Can'tell | Can'tell | No | Yes | Yes | No | Yes | 7 |
| 18 | Psilopanagioti et al. (2012)[34] | Yes | Yes | Yes | Yes | Yes | No | Yes | Yes | Yes | No | Yes | 9 |
| 19 | Rosta and Gerber (2008)[35] | Yes | Yes | Yes | Yes | Can'tell | Can'tell | No | Yes | Can'tell | No | Yes | 6 |
| 20 | Schmit Jongbloed et al. | Yes | Yes | Yes | No | Yes | Can'tell | No | Yes | Yes | No | Yes | 7 |

| | | | | | | | | | | | | | |
|---|---|---|---|---|---|---|---|---|---|---|---|---|---|
| | (2014)[36] | | | | | | | | | | | | |
| 21 | Strömgren et al. (2016)[37] | Yes | Yes | Yes | No | Yes | Can'tell | No | Yes | Yes | No | Yes | 7 |
| 22 | Szilvia et al. (2009)[38] | Yes | Yes | Yes | Yes | Can'tell | No | No | Yes | Yes | No | Yes | 7 |
| 23 | Tartas et al. (2011)[39] | Yes | Yes | Yes | Yes | Yes | No | Can'tell | Yes | Yes | No | Yes | 8 |
| 24 | Visser et al. (2003)[40] | Yes | Yes | Yes | Yes | Can'tell | Can'tell | No | Yes | Yes | Yes | Yes | 8 |

**Supplementary file 4**

**Factors affecting physician satisfaction - analyzed in include studies**

 - statistically significant association  - statistically not significant association

## 1. PERSONAL FACTORS

***Physician age***

| Study ID | Type of measure | Main value | Low range | High range | p value reported | Subgroup/ Comment |
|---|---|---|---|---|---|---|
| Mache et al.[27] | β | -0.02 | NR | NR | >0.05 | NA |
| | R | -0.10 | NR | NR | >0.05 | NA |
| Ommen et al.[32] | B | -0.12 | 0.01 | NR | 0.11 | NA |
| | β | -0.02 | 0.44 | NR | 0.11 | NA |
| Psilopanagioti et al.[34] | B | 0.41 | -2.21 | 3.04 | 0.76 | Self- Emotion Appraisal |
| | β | 0.03 | NR | NR | | |
| | B | 0.46 | -1.96 | 2.87 | 0.71 | Use of Emotion |
| | β | 0.03 | NR | NR | | |
| Heponiemi et al.[22] | β | 0.11 | NR | NR | <0.001 | 117 |
| | F | 9.80 | NR | NR | 0.002 | 38 |
| Rosta and Gerber[35] | β | 0.12 | NR | NR | <0.001 | 326 |
| Bauer and | OR | 1.54 | 0.94 | 2.54 | >0.05 | <35 |

| | | | | | | |
|---|---|---|---|---|---|---|
| Gronenberg[18] | OR | 1.02 | 0.65 | 1.61 | >0.05 | 35-59 |
| | OR | 1.04 | 0.86 | 1.25 | >0.05 | 35-59 |
| | OR | 1.1 | 0.70 | 1.72 | >0.05 | >59 |
| Michinov et al.[31] | β | 0.1 | NR | NR | >0.05 | <36 |
| French et al.[20] | β | 0.002 | NR | NR | NS | staff grade |
| | β | -0.04 | NR | NR | NS | associate specialists |
| Mascia et al.[30] | CC | 0.14 | NR | NR | <0.01 | <36 |
| | CC | −0.01 | NR | NR | NS | 36-40 |
| | CC | 0.12 | NR | NR | NS | 41-45 |
| | CC | -0.06 | NR | NR | NS | =>56 |
| | CC | −0.07 | NR | NR | NS | 46-55 |
| B - unstandardized coefficients; Beta (β) - standardized coefficients; CC - Correlation coefficients, NA – not applicable, NR – not reported, NS – not significant | | | | | | |

**Gender**

| **Study ID** | **Type of measure** | **Reference group** | **Main value** | **Low range** | **High range** | **p value reported** | **Subgroup/ Comment** |
|---|---|---|---|---|---|---|---|
| French et al.[20] | β | F | -0.05 | NR | NR | NS | staff grades |
| | β | F | 0.27 | NR | NR | NS | associate specialists |
| Mache [28] | r | NA | -0.08 | NR | NR | NR | NA |

| | | | | | | | |
|---|---|---|---|---|---|---|---|
| Gaszynska at al.[21] | NR | F | 4.89 | NR | NR | NS | NA |
| Rosta and Gerber[35] | β | F | -0.84 | SE | 0.52 | 0.11 | NA |
| | β | F | 0.002 | NR | NR | 0.92 | NA |
| Aalto et al.[17] | NR | F | -0.05 | NR | NR | <0.01 | NA |
| Bauer and Gronenberg[18] | OR | F | 1.98 | 1.18 | 3.32 | 0.01 | NA |
| | OR | M | 1.11 | 0.88 | 1.39 | NS | NA |
| Psilopanagioti at al.[34] | B | NA | -1.95 | −5.53 | 1.64 | 0.29 | Self- Emotion Appraisal |
| | β | NA | -0.09 | NR | NR | | |
| | B | NA | −2.55 | −5.86 | 0.76 | | Use of Emotion |
| | β | NA | -0.11 | NR | NR | | |
| Heponiemi at al.[22] | β | F | 0 | NR | NR | 0.824 | NA |
| | F | NA | 0.20 | NR | NR | 0.664 | NA |
| Schmit Jongbloed et al.[36] | B | NA | 0.16 | NR | NR | 0.002 | NA |
| | β | NA | 0.19 | NR | NR | 0.002 | NA |
| Michinov et al.[31] | β | NA | 0.11 | NR | NR | >0.05 | NA |
| | r | F | -0.18 | NR | NR | <0.05 | NA |
| Mascia et al.[30] | SE | | 0.06 | 0.21 | NR | NS | NA |
| Mache et al.[27] | β | NA | -0.03 | NR | NR | >0.05 | NA |
| | R | NA | -0.07 | NR | NR | >0.05 | NA |
| Ommen et al.[32] | B | NA | -0.13 | 0.13 | NR | 0.31 | NA |
| | β | NA | -0.05 | 0.94 | NR | 0.31 | NA |

| |
|---|
| B - unstandardized coefficients; Beta (β) - standardized coefficients; F – female, M - male, NA – not applicable, NR – not reported, NS – not significant |

***Gender/Years of practice (interaction)***

| Study ID | Type of measure | Main value | Low range | High range | p value reported |
|---|---|---|---|---|---|
| Schmit Jongbloed et al.[36] | β | -0.02 | NR | NR | NS |
| | B | -0.02 | NR | NR | 0.734 |
| B - unstandardized coefficients; Beta (β) - standardized coefficients, NR – not reported, NS – not significant | | | | | |

***Years of experience***

| Study ID | Definition | Type of measure | Main value | Low range | High range | p value reported |
|---|---|---|---|---|---|---|
| Mache et al.[28] | years of experience | β | -0.14 | NR | NR | <0.05 |
| | | R | -0.15 | NR | NR | NR |
| Michinov et al.[31] | tenure in the team and job satisfaction | Rho | 0.18 | NR | NR | 0.05 |
| | tenure in the team and identification with team | r | 0.18 | NR | NR | <0.05 |
| | tenure | β | 0.12 | NR | NR | >0.05 |
| Schmit Jongbloed et al.[36] | years of practice | B | -0.03 | NR | NR | >0.05 |
| | | β | -0.03 | NR | NR | 0.66 |

| | | | | | | |
|---|---|---|---|---|---|---|
| Gaszynska et al.[21] | =<10 years since graduation | NR | 5.31 | NR | NR | NS |
| | 11-20 years since graduation | NR | 5.04 | NR | NR | NS |
| | 21-30 years since graduation | NR | 4.57 | NR | NR | NS |
| | >30 years since graduation | NR | 4.83 | NR | NR | NS |
| Mascia et al.[30] | tenure | corr ceof | −0.14 | NR | NR | <0.01 |
| | | B | −0.04 | SE | 0.01 | ≤0.01 |
| B - unstandardized coefficients; Beta (β) - standardized coefficients, NR – not reported, NS – not significant | | | | | | |

***Marital status or having a partner***

| Study ID | Definition | Subgroup | Type of measure | Main value | Low range | High range | p value reported |
|---|---|---|---|---|---|---|---|
| French et al.[20] | partner (yes) | Staff | β | 0.25 | NR | NR | NS |
| | family effect | | β | -0.11 | NR | NR | NS |
| | partner (yes) | Assoc specialists | β | 0.18 | NR | NR | NS |
| | family effect | | β | -0.19 | NR | NR | NS |
| Gaszynska et | marital status | single | NR | 4.89 | NR | NR | NS |

| | | | | | | | |
|---|---|---|---|---|---|---|---|
| al.[21] | | married | NR | 4.85 | NR | NR | NS |
| Beta (β) - standardized coefficients; NR – not reported, NS – not significant | | | | | | | |

***Work-family conflict***

| Study ID | Definition | Type of measure | main value | low range | high range | p value reported |
|---|---|---|---|---|---|---|
| Szilvia et al.[38] | work-family conflict all | β | –0.14 | –0.22 | –0.04 | <0.01 |
| | female - work-family conflict | β | –0.17 | –0.31 | –0.04 | <0.05 |
| Heponiemi et al.[22] | work interference with family | B | -0.1 | NR | NR | <0.001 |
| B - unstandardized coefficients; Beta (β) - standardized coefficients, NA – not applicable, NR – not reported | | | | | | |

***Health status***

| Study ID | Definition | Subgroup | Type of measure | Main value | Low range | High range | p value reported |
|---|---|---|---|---|---|---|---|
| French et al.[20] | health state (excellent) | staff | β | 0.49 | NR | NR | <0.05 |
| | | assoc specialist | β | 0.43 | NR | NR | NS |
| Heponiemi et al.[22] | sleeping problems | NA | β | -0.12 | NR | NR | <0.001 |
| Laubach and Fischbeck[26] | personal health | NA | β | -0.19 | NR | NR | <0.05 |

| |
|---|
| Beta (β) - standardized coefficients; NA – not applicable, NR – not reported, NS – not significant |

***Life satisfaction***

| Study ID | Definition | Type of measure | Main value | Low range | High range | p value reported |
|---|---|---|---|---|---|---|
| Rosta and Gerber[35] | life satisfaction | β | 0.56 | NR | NR | <0.05 |
| Beta (β) - standardized coefficients, NR – not reported | | | | | | |

***Coping strategies/psychological construct***

| Study ID | Definition | Type of measure | main value | Low range | High range | p value reported | Subgroup/ Comment |
|---|---|---|---|---|---|---|---|
| Psilopanagioti et al.[34] | surface acting | β | -0.44 | NR | NR | <0.001 | model with Self-Emotion Appraisal as one of the predictors |
| | | B | -1.14 | -1.56 | -0.72 | <0.001 | |
| | self-emotion appraisal | B | 0.01 | -0.42 | 0.44 | NS | |
| | | β | 0.003 | NR | NR | NS | |
| | surface acting | β | -0.42 | NR | NR | <0.001 | model with Use of Emotion as one of the predictors |
| | | B | −1.07 | −1.45 | -0.69 | <0.001 | |
| | use of emotion | B | 0.89 | 0.5 | 1.29 | <0.001 | |
| | | β | 0.33 | NR | NR | <0.001 | |
| Kinzl et al.[25] | satisfactory | corr | 0.25 | | | 0.005 | NA |

| | | | | | | | |
|---|---|---|---|---|---|---|---|
| | emotional well-being | coef | | | | | |
| Visser et al.[40] | communicative responsiveness | OR | 1.39 | 1.2 | 1.61 | <0.05 | NA |
| Tartas et al.[39] | sense of coherence | R2 | 0.40 | NR | NR | <0.05 | NA |
| | depression | R2 | 0.12 | NR | NR | <0.05 | NA |
| | anxiety (state and trait) | R2 | 0.17 | NR | NR | <0.01 | NA |
| | coping styles | R2 | 0.69 | NR | NR | <0.01 | NA |
| Aalto et al.[17] | optimism | Bivariate correlations | 0.34 | NR | NR | <0.01 | NA |
| B - unstandardized coefficients; Beta (β) - standardized coefficients; NA – not applicable, NR – not reported, NS – not significant | | | | | | | |

***Being a foreign physician***

| **Study ID** | **Definition** | **Reference group** | **Type of measure** | **Main value** | **Low range** | **High range** | **p value reported** |
|---|---|---|---|---|---|---|---|
| Peña-Sánchez et al.[33] | internationally trained physicians (MD training outside of Spain) | Locally trained | Exp beta (OR) | 7.81 | 1.4 | 43.48 | <0.05 |

**II. INTRINSIC FACTORS**

***Medical specialty***

| Study ID | Definition | Reference group | Type of measure | Main value | Low range | High range | p value reported |
|---|---|---|---|---|---|---|---|
| Laubach& Fischbeck[26] | non-surgical/ conservative, surgical, clinical-theoretical | NA | β | 0.08 | NR | NR | NR |
| Aalto et al.[17] | specialty was coded 0 for other medical specialists and 1 for psychiatrists | NA | β | -0.06 | NR | NR | <0.01 |
| Heponiemi et al. [22] | Specialization was coded: 0 = not specialized; 1 = specialized or specialization on-going | NA | β | 0.05 | NR | NR | 0.007 |
| French eat al.[20] | general surgery | SG | β | -0.31 | NR | NR | NR |
| | | AS | β | 0.70 | NR | NR | NR |
| | anaesthetics | SG | β | 0.45 | NR | NR | NR |
| | | AS | β | 0.47 | NR | NR | NR |
| | paediatrics | SG | β | -0.11 | NR | NR | NR |
| | | AS | β | 1.57 | NR | NR | NR |
| | laboratory medicine and radiology | SG | β | 1.51 | NR | NR | NR |
| | | AS | β | 0.08 | NR | NR | NR |
| | psychiatry | SG | β | -0.04 | NR | NR | NR |

| | | | | | | | |
|---|---|---|---|---|---|---|---|
| | | AS | β | 1.06 | NR | NR | NR |
| | obstetrics & gynaecology | SG | β | 0.41 | NR | NR | NR |
| | | AS | β | 1.04 | NR | NR | NR |
| | oral medicine | SG | β | -0.65 | NR | NR | NR |
| | | AS | β | -0.72 | NR | NR | NR |
| | accident & emergency | SG | β | -0.4 | NR | NR | NR |
| | | AS | β | 2.53 | NR | NR | NR |
| AS - associate specialist; Beta (β) - standardized coefficients; NA – not applicable, NR – not reported, NS – not significant, SG – staff grade | | | | | | | |

***Patients interactions***

| **Study ID** | **Definition** | **Type of measure** | **Main value** | **Low range** | **High range** | **p value reported** |
|---|---|---|---|---|---|---|
| Mache et al.[28] | patients' overall satisfaction | r | 0.49 | NR | NR | <0.05 |
| Janus et al.[23] | patient contact | Pearson correlation | 0.117 | NR | NR | <0.05 |
| NR – not reported | | | | | | |

***Work engagement***

| **Study ID** | **Definition** | **Type of measure** | **Main value** | **Low range** | **High range** | **p value reported** |
|---|---|---|---|---|---|---|
| Mache et al.[29] | work engagement | Regr coef adj | 0.24 | NR | NR | <0.01 |
| | | Pearson r | 0.48 | NR | NR | <0.01 |

| | | | | | | |
|---|---|---|---|---|---|---|
| | work engagement - vigor | Pearson r | 0.46 | NR | NR | <0.01 |
| | work engagement - dedication | Pearson r | 0.45 | NR | NR | <0.01 |
| | work engagement - absorption | Pearson r | 0.41 | NR | NR | <0.01 |
| NR – not reported | | | | | | |

### *III.* CONTEXTUAL FACTORS: WORK-PLACE RELATED

***Hospital type and structure***

| Study ID | Definition | Subgroup | Type of measure | Main value | Low range | High range | p value reported |
|---|---|---|---|---|---|---|---|
| Heponiemi et al.[22] | private employer | * | β | 0.09 | NR | NR | <0.001 |
| | | job control* | β | 0.04 | NR | NR | 0.013 |
| | | Interpersonal justice* | β | 0.04 | NR | NR | 0.076 |
| | | Informational justice* | β | 0.04 | NR | NR | 0.079 |
| | | Distributional justice* | β | 0.03 | NR | NR | NR |
| | | Coded 0=public employer, 1=private employer | β | 0.08 | NR | NR | <0.001 |
| Beta (β) - standardized coefficients, NR – not reported, * adjusted for gender, age, specialization, part-time employment | | | | | | | |

***Management/Leadership***

| Study ID | Definition | Type of measure | Main value | Low range | High range | p value reported |
|---|---|---|---|---|---|---|
| van Beuzekom et al.[19] | planning and coordination: 'Lack of advance planning within the department' | β | -0.41 | NR | NR | <0.001 |
| | | BC | -0.7 | NR | NR | NR |
| | | BC | -0.36 | NR | NR | NR |
| | Hierarchy: 'In my department, staff do not always dare to ask for an explanation | β | -0.26 | NR | NR | <0.01 |
| | | BC | -0.64 | NR | NR | <0.05 |
| | | BC | -0.40 | NR | NR | <0.05 |
| | Maintenance: 'Maintenance inspections are carried out on time' | BC | -0.28 | NR | NR | <0.05 |
| | | BC | -0.4 | NR | NR | <0.05 |
| | | β | -0.39 | NR | NR | <0.05 |
| Strömgren et al.[37] | trust regarding management | NR | 0.11 | NR | NR | <0.000 |
| Laubach& Fischbeck[26] | superiors and hierarchy (FAÄ-V) | β | -0.49 | NR | NR | NR |
| Heponiemi et al.[22] | procedural justice | PR | 0.24 | NR | NR | < 0.05 |
| | | PR | 0.29 | NR | NR | < 0.05 |
| | interactional justice | PR | 0.2 | NR | NR | < 0.05 |
| | | PR | 0.21 | NR | NR | < 0.05 |
| | distributive justice | PR | 0.19 | NR | NR | < 0.05 |

| | | | | | | |
|---|---|---|---|---|---|---|
| | | PR | 0.17 | NR | NR | < 0.05 |
| | organizational justice | β | 0.39 | NR | NR | <0.001 |
| Visser et al.[40] | feeling poorly managed and resourced | OR | 2.07 | 1.76 | 2.43 | <0.05 |
| Mache et al.[27] | quality of leadership =what extent would you say that your immediate superiors are good in solving conflicts | β | 0.16 | NR | NR | <0.01 |
| | | R | 0.52 | NR | NR | <0.01 |
| Jönsson[24] | role conflicts T1 | β | 0.17 | NR | NR | <0.001 |
| | role conflicts T2 | β | 0.23 | NR | NR | <0.001 |
| Mascia et al.[30] | pioneeric change | B | −0.97 | SE | 0.3 | ≤0.01. |
| | | CC | −0.05 | NR | NR | NS |
| | process integration | CC | 0.12 | NR | NR | NS |
| | mixed intergation | CC | 0.11 | NR | NR | NS |
| | teaching hospital | CC | 0.04 | NR | NR | NS |
| | research hospital | CC | 0.22 | NR | NR | p < 0.01 |
| | | B | 0.73 | SE | 0.3 | <0.05 |
| | LHA hospital | B | −0.61 | SE | 0.26 | <0.05 |
| | | CC | −0.24 | NR | NR | <0.01 |
| | hospital trust | B | 0.92 | SE | 0.32 | ≤0.01 |
| | | CC | 0.08 | NR | NR | S |

B - unstandardized coefficients; Beta (β) - standardized coefficients; BC - Bivariate correlations, CC – Correlation coefficients, NR – not reported, NS – not significant, PR – Pearson R, S – significant

***Opportunity for professional development***

| Study ID | Definition | Type of measure | Main value | Low range | High range | p value reported |
|---|---|---|---|---|---|---|
| Mache et al.[27] | possibilities for development "do you have the possibility of learning new things through your work?" | β | 0.21 | NR | NR | <0.001 |
| | | R | 0.44 | NR | NR | <0.01 |
| Janus et al.[23] | continuous education and job security | PC | 0.3 | NR | NR | <0.000 |
| | research and teaching | PC | 0.09 | NR | NR | 0.074 |
| | international exchange | PC | 0.08 | NR | NR | 0.117 |
| Mache et al.[28] | possibilities for development | r | 0.54 | NR | NR | <0.01 |
| | feedback at work | r | 0.41 | NR | NR | <0.05 |
| | | β | 0.05 | NR | NR | >0.05 |
| | | R | 0.26 | NR | NR | <0.01 |
| Visser et al.[40] | intellectual stimulation | OR | 1.38 | 1.17 | 1.62 | <0.05 |
| Mache et al.[29] | possibilities for development | β | 0.2 | NR | NR | <0.05 |
| | | r | 0.43 | NR | NR | <0.05 |
| | feedback | β | 0.05 | NR | NR | >0.05 |
| | | r | 0.25 | NR | NR | <0.05 |

Beta (β) - standardized coefficients, PC – Pearson correlations , NR – not reported

***Colleague support (team work. relations with team)***

| Study ID | Definition | Type of measure | Main value | Low range | High range | p value reported |
|---|---|---|---|---|---|---|
| Strömgren et al.[37] | recognition | NR | 0.32 | NR | NR | <0.000 |
| | increased social capital predicted increased job satisfaction | OR | 2.2 | 1.4 | 3.4 | S |
| | social reciprocity | NR | 0.16 | NR | NR | <0.0000 |
| Visser et al.[40] | social support from colleagues | OR | 1.14 | 1.18 | 1.62 | S |
| Laubach & Fischbeck[26] | team ability in coping with conflicts | β | -0.13 | NR | NR | NR |
| Laubach& Fischbeck[26] | workload cooperation in / organization of the ward | β | -0.13 | NR | NR | NR |
| Kinzl et al.[25] | participation vs satisfaction | r | 0.378 | NR | NR | 0.001 |
| Janus et al.[23] | collegial relationships | PR | 0.19 | NR | NR | <0.000 |
| Jönsson[24] | social supportT1 | β | 0.17 | NR | NR | <0.001 |
| | social supportT2 | β | 0.32 | NR | NR | <0.001 |
| Aalto et al.[17] | team climate | NR | 0.42 | NR | NR | <0.01 |
| Mache et al.[29] | sense of community | β | 0.25 | NR | NR | <0.001 |
| | | R | 0.48 | NR | NR | <0.01 |

| | | | | | | |
|---|---|---|---|---|---|---|
| | | r | 0.49 | NR | NR | <0.01 |
| | social support | β | 0.04 | NR | NR | >0.05 |
| | | R | 0.42 | NR | NR | <0.01 |
| | | r | 0.39 | NR | NR | <0.01 |
| | social relations | β | -0.03 | NR | NR | >0.05 |
| | | R | 0.16 | NR | NR | <0.05 |
| | | r | 0.42 | NR | NR | <0.01 |
| Ommen et al.[32] | social capital | B | 0.16 | SE | 0.02 | NS |
| | | β | 0.45 | NR | NR | NS |
| Michinov et al.[31] | transactive memory system | Rho | NR | NR | NR | 0.43 |
| | size of the team | β | -0.27 | NR | NR | < .001 |
| | coordination | β | 0.34 | NR | NR | <0.001 |
| | tenure in the team and work attitudes | r | 0.21 | NR | NR | <0.01 |
| | tenure in the team and perceptions of team effectiveness | r | 0.21 | NR | NR | <0.01 |
| | team size | r | -0.28 | NR | NR | <0.01 |
| | credibility | β | 0.14 | NR | NR | <0.001 |
| B - unstandardized coefficients; Beta (β) - standardized coefficients; NR – not reported, NS – not significant, S – significant, PC – Pearson correlations | | | | | | |

***Access to resources***

| **Study ID** | **Definition** | **Reference group** | **Type of measure** | **Main value** | **Low range** | **High range** | **p value reported** |
|---|---|---|---|---|---|---|---|
| van Beuzekom et al.[19] | material resources | anesthetists | Bivariate correlations | -0.35 | NR | NR | <0.05 |
| | | trainee anesthetists | Bivariate correlations | -0.17 | NR | NR | NS |
| Janus et al.[23] | specialized technology | NA | Pearson correlation | 0.13 | NR | NR | <0.05 |
| NA – not applicable, NR – not reported, NS – not significant | | | | | | | |

## III. CONTEXTUAL FACTORS: JOB RELATED

***Workload and job demand***

| **Study ID** | **Definition** | **Type of measure** | **Main value** | **Low range** | **High range** | **p value reported** |
|---|---|---|---|---|---|---|
| Heponiemi et al.[22] | distress at first examination | PR | -0.21 | NR | NR | < 0.05 |
| | distress at second examination | PR | -0.21 | NR | NR | < 0.05 |
| Visser et al.[40] | stress | r | –0.25 | NR | NR | 0.001 |
| Aalto et al.[17] | psychological distress | BC | -0.43 | NR | NR | <0.01 |
| BC - Bivariate correlations; NR – not reported, NS – not significant, PR - Pearson R | | | | | | |

***Work control***

| **Study ID** | **Definition** | **Reference group** | **Type of measure** | **Main value** | **Low range** | **High range** | **p value reported** |
|---|---|---|---|---|---|---|---|
| French et | equal member | staff | β | 1.01 | NR | NR | <0.05 |

| | | | | | | | |
|---|---|---|---|---|---|---|---|
| al.[20] | equal member | assoc specialist | β | 1.42 | NR | NR | <0.05 |
| Mache et al.[28] | influence at work | NA | r | 0.40 | NR | NR | <0.05 |
| | degree of freedom | NA | r | 0.27 | NR | NR | <0.01 |
| B - unstandardized coefficients; Beta (β) - standardized coefficients, NA – not applicable, NR – not reported | | | | | | | |

***Work stability***

| Study ID | Definition | Reference group | Type of measure | Main value | Low range | High range | p value reported |
|---|---|---|---|---|---|---|---|
| Heponiemi et al.[22] | part-time employment | Coded 0 = full-time employment. 1 = part-time employment | β | -0.05 | NR | NR | 0.012 |
| Visser et al.[40] | job security | NA | OR | 1.65 | 1.39 | 1.95 | <0.05 |
| B - unstandardized coefficients; Beta (β) - standardized coefficients, NA – not applicable, NR – not reported | | | | | | | |

***Being a chief***

| Study ID | Definition | Reference group | Type of measure | Main value | Low range | High range | p value reported |
|---|---|---|---|---|---|---|---|

| | | | | | | | |
|---|---|---|---|---|---|---|---|
| Rosta and Gerber [35] | 0=chief. 1-assistance | NA | β | -4.97 | SE | 0.66 | 0.0001 |
| Bauer&Gronenberg[18] | being chief doctor | Medical Assistants | OR | 3.50 | 1.94 | 6.32 | S |
| Mascia et al.[30] | 1=no role 2=semplice 3=compless a | no managerial role | CC | -0.1 | NR | NR | NS |
| | | manag. role "Str Complessa" | B | −0.19 | SE | 0.4 | NS |
| | | | CC | 0.06 | NR | NR | NS |
| | | manag. role "Str Semplice" | CC | 0.07 | NR | NR | NS |
| | | | B | 0.55 | SE | 0.84 | NS |
| B - unstandardized coefficients; Beta (β) - standardized coefficients, CC - Correlation coefficients, NA – not applicable, NR – not reported, NS – not significant | | | | | | | |

***Income***

| **Study ID** | **Definition** | **Reference group** | **Type of measure** | **Main value** | **Low range** | **High range** | **p value reported** |
|---|---|---|---|---|---|---|---|
| French et al. [20] | £20,000–£34,999 per year | staff grade | β | 0.28 | NR | NR | NS |
| | £20,000–£34,999 per year | associate specialist | β | 0.16 | NR | NR | NS |
| | £35,000–£49,999 per year | staff grade | β | 0.53 | NR | NR | S |
| | £35,000–£49,999 per year | associate specialist | β | 1.07 | NR | NR | NS |

| | | | | | | | |
|---|---|---|---|---|---|---|---|
| | >£49,999 per year | staff grade | β | 0.61 | NR | NR | NS |
| | >£49,999 per year | associate specialist | β | 0.83 | NR | NR | NS |
| Beta (β) - standardized coefficients, NR – not reported, NS – not significant, S – significant | | | | | | | |

***Non-financial incentives***

| Study ID | Definition | Reference group | Type of measure | Main value | Low range | High range | p value reported |
|---|---|---|---|---|---|---|---|
| Peña-Sánchez eat al.[33] | having received non-monetary incentives during last year | Have not received non-monetary incentives | Exp beta (OR) | 3.11 | 1.19 | 8.13 | <0.05 |
| Visser et al.[40] | feeling valued | NA | OR | 1.44 | 1.19 | 1.75 | <0.05 |
| NA – not applicable | | | | | | | |

**<u>III. CONTEXTUAL FACTORS: OTHER</u>**

***Intension to leave***

| Study ID | Definition | Subgroup | Type of measure | Main value | Low range | High range | p value reported |
|---|---|---|---|---|---|---|---|
| van Beuzekom et al.[19] | intention to leave | *anesthetists* | BC | -0.61 | NR | NR | <0.05 |
| | | *trainees* | BC | 0.42 | NR | NR | <0.01 |
| BC - Bivariate correlations, NR – not reported | | | | | | | |

***Prior achievement***

| **Study ID** | **Definition** | **Type of measure** | **Main value** | **Low range** | **High range** | **p value reported** |
|---|---|---|---|---|---|---|
| Schmit Jongbloed et al.[36] | curriculum (Traditional vs PBL) | β | 0.04 | NR | NR | NS |
| Beta (β) - standardized coefficients;  NR – not reported, NS – not significant | | | | | | |
